# Non drowsy obstructive sleep apnea as a potential cause of resistant hypertension: a case report

**DOI:** 10.1186/1471-2466-12-16

**Published:** 2012-05-17

**Authors:** Aibek E Mirrakhimov

**Affiliations:** 1 Kyrgyz State Medical Academy named after I.K. Akhunbaev, Akhunbaev street 92, Bishkek, 720020, Kyrgyzstan; 2 National Centre of Cardiology and Internal Medicine named after M. Mirrakhimov, T.Moldo 3, Bishkek, 720040, Kyrgyzstan

**Keywords:** Sleep Apnea, Arterial Hypertension, Resistant Hypertension, Obesity

## Abstract

**Background:**

Obstructive sleep apnea (OSA) and arterial hypertension (AH) are common and underrecognized medical disorders. OSA is a potential risk factor for the development of AH and/or may act as a factor complicating AH management. The symptoms of excessive daytime sleepiness (EDS) are considered essential for the initiation of continuous positive airway pressure (CPAP) therapy, which is a first line treatment of OSA. The medical literature and practice is controversial about the treatment of people with asymptomatic OSA. Thus, OSA patients without EDS may be left at increased cardiovascular risk.

**Case presentation:**

The report presents a case of 42year old Asian woman with symptoms of heart failure and angina like chest pain upon admission. She didnt experience symptoms of EDS, and the Epworth Sleepiness Scale was seven points. Snoring was reported on direct questioning. The patient had prior medical history of three unsuccessful pregnancies complicated by gestational AH and preeclampsia with C-section during the last pregnancy. The admission blood pressure (BP) was 200/120mm Hg. The patients treatment regimen consisted of five hypotensive medications including diuretic. However, a target BP wasnt achieved in about one and half month. The patient was offered to undergo a polysomnography (PSG) study, which she rejected. One month after discharge the PSG study was done, and this showed an apnea-hypopnea index (AHI) of 46 events per hour. CPAP therapy was initiated with a pressure of 11H_2_0cm. After 2months of compliant CPAP use, adherence to pharmacologic regimen and lifestyle modifications the patients BP decreased to 134/82mm Hg.

**Conclusions:**

OSA and AH are common and often underdiagnosed medical disorders independently imposing excessive cardiovascular risk on a diseased subject. When two conditions coexist the cardiovascular risk is likely much greater. This case highlights a possible clinical phenotype of OSA without EDS and its association with resistant AH. Most importantly a good hypotensive response to medical treatment in tandem with CPAP therapy was achieved in this patient. Thus, it is reasonable to include OSA in the differential list of resistant AH, even if EDS is not clinically obvious.

## Background

Obstructive sleep apnea (OSA)-is the disorder characterized by complete or partial breathing disturbances during sleep with a minimum prerequisite frequency of 5 events per hour and lasting for at least 10 seconds [1]. The prevalence of OSA varies in epidemiological surveys, and this can be explained by different populations studied and including excessive daytime sleepiness (EDS) as a criterion for OSA diagnosis [2]. Thus, it is likely that the real number of the affected population is much higher than reported.

OSA is associated with increased cardiovascular risk and OSA in particular may be an independent risk factor for the development of arterial hypertension (AH) [3]. AH on the other hand, is a major cardiovascular risk factor and among the most prevalent chronic conditions worldwide [4]. The association between AH and OSA without symptoms of EDS is conflicting and there are controversies regarding the place of continuous positive airway pressure (CPAP) therapy in such situations. Thus, non-sleepy OSA individuals may be dishonestly left with increased cardiovascular risk.

## Case presentation

A 42year old female of Asian descent was admitted to the ward with complaints of dyspnea and squeezing chest pain without radiation during mild to moderate physical activity, pitting edema of the lower extremities, nocturia and treatment resistant AH. For the last 6months, the patient experienced shortness of breath and lower extremities pitting edema which had worsened with time. During this period, the patient reported fatigue, which was related to the aforementioned symptoms from the patients own words. Upon questioning the patient reported loud snoring during sleep, but denied sleepiness during the wake time.

The patient is Gravida 3 Para 0. The first 2 pregnancies were complicated with gestational hypertension (which were resolved after pregnancies) with stillbirths and the last one with preeclampsia and emergent C-section delivery of demised infant at 30weeks term.

AH was diagnosed at 2006, during regular outpatient visit with measured blood pressure (BP) 186/110mm Hg at that time. Since then, the patient noticed angina like chest pain during regular physical activity. Family history is remarkable for obesity and AH in both of her parents.

The patients prehospitalization regimen consisted of 100mg of atenolol a day and 20mg of enalapril a day, which is not believed to be an optimal hypotensive combination therapy [5]. The patients BP ranged from 160/100mm Hg to 240/130mm Hg (which is the highest retrospectively recorded BP in this patient). The patient denied smoking, alcohol intake or use of any psychostimulating (including caffeine containing products) remedies.

The patient is obese for the last 15years, but since 2009 she gained approximately 10 kilograms. The patient ate fatty meals with average daily calorie intake of approximately 3000 Kcal/day and followed sedentary lifestyle. The body weight was 103kg, height was 156cm, abdominal circumference was 134cm and body mass index was 42.3kg/m^2^ upon admission.

Cardiovascular examination: heart rate (HR) was 85 beats per minute. The loud second heart sound was heard over the right second intercostal space. No murmurs, rubs or gallops upon auscultation were heard. Bilateral pitting edema was present over the shins. Admission BP was 200/120mm Hg.

On the pulmonary exam, bilateral inspiratory rales were present at the bases with no change on coughing. The respiratory rate was 19 per minute. Digital pulse oximetry revealed oxygen saturation of 95%.

Oral examination revealed redundant pharyngeal soft tissue and Mallampati class 3 [6]. Neck circumference was 43cm the thyroid gland wasnt palpable. No hair loss, skin changes or alterations in bowel habits were present. Neurological exam was intact. The Epworth sleepiness score (ESS) was seven points [7].

Complete blood count, Creatinine, electrolytes, glomerular filtration rate, liver function tests (ALT, AST), troponin level, fasting lipid panel, fasting glucose (on 2 separate occasions) and thyroid function tests were all within normal limits.

Electrocardiography (ECG): inverted T waves in leads V3-V6. Holter ECG monitoring: 1 paroxysm of atrial fibrillation with HR of 134 beats/minute lasting for 21 seconds. Frequent episodes of T wave inversion without overt ST segment abnormalities.

Echocardiography: left ventricular end diastolic dimension: 6.45cm, left ventricular end systolic dimension: 5.01cm, interventricular septum: 1.36cm, posterior wall of the left ventricle: 1.27cm and ejection fraction of 43%. Mild diffuse left ventricular hypokinesis was present. Other parameters were within normal limits.

Carotid ultrasound: left common carotid intima media thickness of 0.91 and right common carotid TIM of 0.92 without obvious plaques.

24 hour BP measurement: non dipping pattern, with only 3% of nighttime decline of BP. Important to note that nighttime BP is known to be a stronger predictor of cardiovascular events than daytime BP [8].

Chest X-Ray: Pulmonary venous congestion and cardiothoracic index of 49%.

The hospital stay was 20days. The patients hypotensive regimen is present in Table 1. On this regimen the patients BP ranged from 150/90 to170/110mm Hg. HR was 6270 beats per minute. Besides the pharmacological intervention, the patient was counseled on proper low fat/calorie diet and other measures to improve her lifestyle.

**Table 1:** Hypotensive regimen at the hospital

**Medication**	**Dose**
Bisoprolol	10mg a day
Candesartan	32mg a day
Indapamide SR	1.5mg a day
Amlodipine	10mg a day
Moxonidine	400 mcg a day

The patient was considered to have resistant AH. Since this patient was obese and had high diastolic BP some possible alternative etiologies were considered such as obesity related AH, hypothyroidism, OSA etc.

Since OSA was in the differential list, this patient was offered a PSG study, which she rejected at that time.

The patient was discharged with the BP of 160/106mm Hg and instructed to return for follow up visit in 2weeks as outpatient.

The patient arrived only 1month after and assured that she didnt have any compliance related problems. The BP was 158/110mm Hg. Dyspnea became less severe and the angina episodes less frequent.

**Figure 1:**
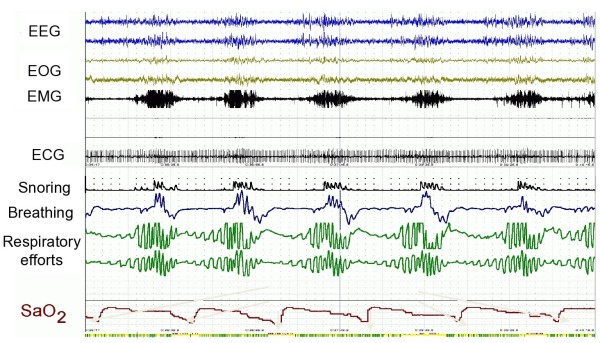
**Patients PSG recording.Abbreviations**: **ECG**-electrocardiography, **EEG**-electroencephalography, **EMG**-electromyography, **EOG**-electrooculography, **SaO2**-oxygen saturation.

The possibility of OSA was again discussed with the patient and PSG study was offered, which was agreed at that time. The recording of the patients PSG study is present in Figure 1.

The 12 hour PSG revealed the apnea-hypopnea index (AHI) of 46 events per hour, which is consistent with a severe form of the disease [1]. On the next night, the CPAP titration study with the pressure of 11H_2_0cm abolished the sleep disordered breathing events. The patient was instructed on proper and compliant use of CPAP machine. Follow up was scheduled in 2months.

After 2months, the patients CPAP using time was on average 6 hours per night and 7days a week. The BP on this visit was 140/92mm Hg on prior pharmacological regimen and CPAP therapy.

One month later, the patients BP became 134/82mm Hg. The patients treatment regimen wasnt modified during this time interval. Home sleep monitoring detected the AHI of six events per hour.

## Discussion

Resistant AH is defined as inability to reach target BP using at least 3 different hypotensive medications including diuretic [9]. The differential list is broad and includes: lack of treatment compliance, inadequate treatment regimen, hypervolemic states, identifiable causes of AH, obesity and OSA [10].

It is well known that OSA is a potential risk factor for AH development and/or a factor complicating its treatment. Currently the symptoms of EDS are considered essential for the initiation of CPAP therapy, while patients with pathologic AHI and without EDS are generally not treated. However, it is noteworthy to mention that OSA with EDS has the strongest association with resistant AH [11].

In their landmark study, Barbe and colleagues highlighted the beneficial effects of CPAP therapy in non-sleepy OSA subjects who were compliant with CPAP therapy. However in their study the effect on BP was only evident after one year of regular CPAP therapy [12].

In this patient several issues should be considered. First, this is only a case report with all potential limitations. Second, the impact of obesity and insulin resistance werent measured in the patient, but it is important to mention that the patient didnt lose weight within the 3months follow up. Third, the patient decreased her sodium intake from eight grams to four grams a day and started mild to moderate aerobic physical exercises. Fourth, the BP decline may be solely explained by hypotensive effect of BP lowering medications. Fifth, ESS is known to have some limitations, since it is a subjective test for EDS [13]. A recent study highlighted the fact that patients with OSA and concomitant heart failure (HF) can present without symptoms of EDS assessed with ESS [14]. However, others in a smaller number of recruited participants have failed to show that patients with OSA and HF have less EDS [15]. But, on the other hand, ESS may have limited sensitivity for detecting EDS in patients with OSA and concomitant HF.

Nevertheless, this report highlights the possibility of a distinct non sleepy clinical phenotype of OSA and its association with resistant AH. Thus, it is reasonable to include OSA in the differential list of refractory to treatment AH, even when the EDS is not clinically obvious.

## Conclusion

OSA and AH are common and often underdiagnosed medical disorders independently imposing excessive cardiovascular risk on diseased subject. When two conditions coexist the burden is likely much greater. This case highlights a possible association between non-sleepy OSA and AH, with good hypotensive response to BP lowering medications in tandem with CPAP therapy. Thus, it is reasonable to include OSA in the differential list of refractory to treatment AH, even when the EDS is not clinically obvious.

## Consent

Written informed consent was obtained from the patient for publication of this Case report and any accompanying images. A copy of the written consent is available for review by the Series Editor of this journal.

## Abbreviations

AH = Arterial hypertension; AHI = Apnea hypopnea index; BP = Blood pressure; CPAP = Continuous positive airway pressure; ECG = electrocardiography; EDS = Excessive daytime sleepiness; ESS = Epworth sleepiness score; HF = Heart failure; HR = Heart rate; OSA = Obstructive sleep apnea; PSG = Polysomnography.

## Competing of interest

The author declare that he has no competing interests.

## Pre-publication history

The pre-publication history for this paper can be accessed here:

http://www.biomedcentral.com/1471-2466/12/16/prepub
